# miR-223-3p inhibits the progression of atherosclerosis via down-regulating the activation of MEK1/ERK1/2 in macrophages

**DOI:** 10.18632/aging.203908

**Published:** 2022-02-24

**Authors:** Daofeng You, Qiuge Qiao, Katsushige Ono, Mei Wei, Wenyun Tan, Cuihua Wang, Yangong Liu, Gang Liu, Mingqi Zheng

**Affiliations:** 1 Hebei Medical University First Affiliated Hospital, Shijiazhuang 050023, Hebei, China; 2 Hebei Medical University Second Affiliated Hospital, Shijiazhuang 050023, Hebei, China; 3 Department of Pathophysiology, Oita University School of Medicine, Hasama, Yufu, Ōita-shi, Japan

**Keywords:** atherosclerosis, MEK1/ERK1/2 signaling pathway, inflammatory response, macrophages, miR-223-3p

## Abstract

Background: microRNAs (miRNAs) have drawn more attention to the progression of atherosclerosis (AS), due to their noticeable inflammation function in cardiovascular disease. Macrophages play a crucial role in disrupting atherosclerotic plaque, thereby we explored the involvement of miR-223-3p in the inflammatory response in macrophages.

Methods: RT-qPCR was used to analyze the miR-223-3p levels in carotid arteries and serum of AS patients. ROC curve was used to assess the diagnostic value of miR-223-3p. Movat staining was applied to evaluate the morphological differences. FISH was used to identify the expression of miR-223-3p in macrophages of atherosclerotic lesions. Bioinformatic analysis was performed. Double-immunofluorescence and western blot were performed to assess the inflammatory cytokine secretion and p-ERK1/2. C16-PAF was injected into the culture medium of the miR-223-3p mimic/NC-transfected macrophages with ox-LDL.

Results: MiR-223-3p was up-regulated in AS patients and was associated with a higher overall survival rate. MiR-223-3p was co-localized with CD68+ macrophages in vulnerable atherosclerotic lesions. MiR-223-3p mimics decreased atherosclerotic lesions, macrophages numbers whereas increased SMCs numbers in the lesions. The TNF-a immune-positive areas were reduced by miR-223-3p mimics. MAP2K1 was negatively associated with miR-223-3p. MiR-223-3p mimics reduced the inflammation and the MEK1/ERK1/2 signaling pathway *in vivo* and *in vitro*. C16-PAF reversed the effects of miR-223-3p mimics on inflammation and ERK1/2 signaling pathway.

Conclusions: MiR-223-3p negatively regulates inflammatory responses by the MEK1/ERK1/2 signaling pathway. Our study provides new insight into how miR-223-3p protects against atherosclerosis, representing a broader therapeutic prospect for treating atherosclerosis by miR-223-3p.

## INTRODUCTION

Atherosclerosis (AS) is the cause of most cardiovascular disease types, which becomes the primary reason for morbidity and mortality worldwide [1, 2]. Atherosclerotic lesions are characterized by the amplification of inflammatory response and accumulation of lipid droplet [2–4]. Macrophages play an essential role in the immune system and inflammatory reactions, which contribute to the development of atherosclerotic plaques with an astonishing inflammatory dysfunction [5–7].

The Raf/mitogen-activated protein kinase (MEK/MAPKK)/extracellular signal-regulated kinase 1/2 (ERK 1/2) signaling pathway regulates multiple cellular processes such as proliferation, differentiation, and cell growth, and ERK phosphorylation is activated by MEK [8, 9]. On activation, Raf mediates phosphorylation of MEK1 and MEK2, which in turn phosphorylate ERK1 and ERK2, correspondingly [10, 11]. The activation of ERK1/2 signaling may contribute to chronic inflammation, which is involved in arteriosclerosis development [12, 13].

Previous studies have described an association between the modifications in microRNA (miRNA) and atherosclerosis [14–16]. MiRNAs are endogenous non-coding RNAs with a length of approximately 21nt-25nt (nucleotide), which control gene expression and play a critical role in vascular functions and atherosclerosis through post-transcriptional repression [14, 17]. Evidence has indicated that miRNAs target genes are related to chronic inflammation and macrophage activation [18, 19]. Several previous studies highlight that miR-223-3p is a novel prognostic marker in cardiovascular diseases [20, 21], and miR-223-3p can potentially affect inflammatory response and macrophage accumulation [22]. However, the role of miR-223-3p in atherosclerosis disease and its underlying molecular mechanism has not been investigated to date. The miRDIP and starBase elucidate that MEK1 was a target gene of miR-223-3p. Evidence has indicated that the MAPK signaling pathway is involved in miR-223-3p inhibition [23]. However, whether miR-223-3p involves in arteriosclerosis through down-regulating MEK1/ERK1/2 is undiscovered.

Therefore, the present study was conducted to explore the effects and regulatory mechanisms of miR-223-3p /MEK/ERK1/2 in arteriosclerosis. ApoE-/- mice were fed with a high-fat diet (HFD) to induce AS models, macrophages treated with ox-LDL were served as cell AS models.

## RESULTS

### Enhanced MiR-223-3p levels in carotid arteries and serum from patients with AS and co-localization with macrophages in vulnerable atherosclerotic lesion

**Figure 1:**
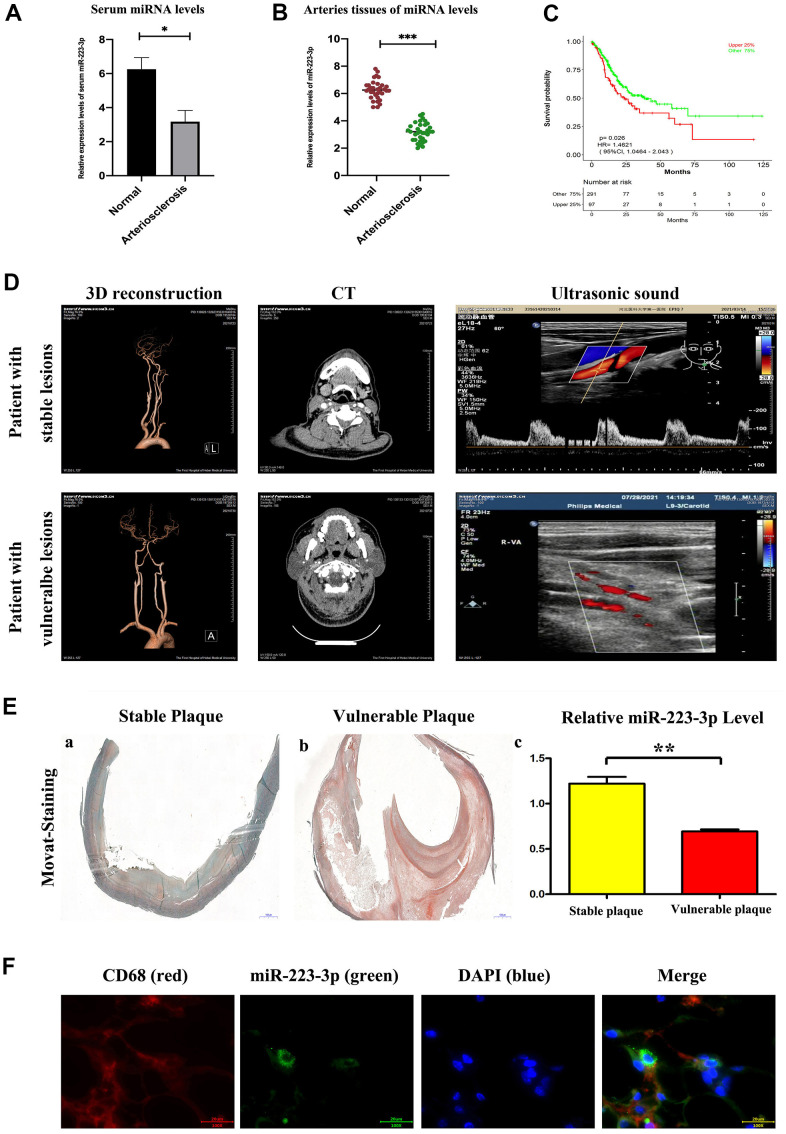
**Enhanced miR-223-3p levels in carotid arteries and serum from patients with AS.** (**A**) The levels of miR-223-3p were higher in AS arteries as compared with normal arteries determined by qPCR. (*P<0.05). (**B**) The levels of miR-223-3p were higher in the serum of AS patients compared with normal controls determined by qPCR. (*P<0.05). (**C**) The expression level of miR-223-3p has been associated with a higher overall survival rate by ROC curve. (**D**) The three-dimensional vascular remodeling, CT, ultrasonic sound pictures from patient with stable or vulnerable lesions were shown. (**E**) Histological analysis on the aortic sections evaluates the morphological differences by Movat staining. Representative staining results for vulnerable plaque and stable plaque. Decreased miR-223-3p in the vulnerable plaque as compared with stable plaque (*P<0.05). (**F**) Fluorescent *In Situ* Hybridization assay revealed miR-223-3p (green) co-localization with CD68+ (red) macrophages in vulnerable atherosclerotic lesions of AS patients. DAPI (blue) for nuclei staining.

To evaluate the expression level of miR-223-3p as potential biomarkers of AS, the information of the patients were shown in Table 1. The qPCR results revealed that the expression level of miR-223-3p was up-regulated in both serum and carotid arteries samples of AS patients compared with controls. (Figure 1A, 1B, P<0.05). Furthermore, we performed the receiver operating characteristic (ROC) curve to assess the diagnostic value of miR-223-3p. We found that the expression level of miR-223-3p is associated with a higher overall survival rate, suggesting that the miR-223-3p could be an excellent diagnostic marker for AS (Figure 1C). And the medical imaging were shown in Figure 1D. Consequently, we carried out a histological analysis on the carotid artery sections to evaluate the morphological differences using Movat staining. Representative staining results for vulnerable plaque and stable plaque were exhibited in Figure 1E.a, b, and we found the vulnerable plaque was associated with increased medial thickness and luminal diameter, and the messages about the patients were shown in Table 1. Additionally, we also found reduced miR-223-3p expression levels in the vulnerable plaque compared with stable plaque (Figure 1E.c). In addition, we carried out a Fluorescent *in situ* Hybridization (FISH) assay to identify the miR-223-3p in macrophages of the intima and vulnerable atherosclerotic lesions. We found miR-223-3p co-localization with CD68+ macrophages in vulnerable atherosclerotic lesions of AS patients (Figure 1F).

**Table 1:** Clinicopathological characteristics of atherosclerotic patients.

**Characteristics**	**Stable group**	**Vulnerable group**
Age	64.25±3.038 N=4	69.50±2.630 N=4
Gender	Male:3/Femal:1	Male:3/Femal:1
Have hyperlipidemia	N=4	N=4
Have diabetes	N=1	N=2
Have hypertension	N=2	N=3
Maximum diameter under ultrasonic(mm)	1.098 ±0.093 N=4	3.283 ?0.2463 N=4**

### Prevention of AS progression and inflammation by miR-223-3p mimics

**Figure 2:**
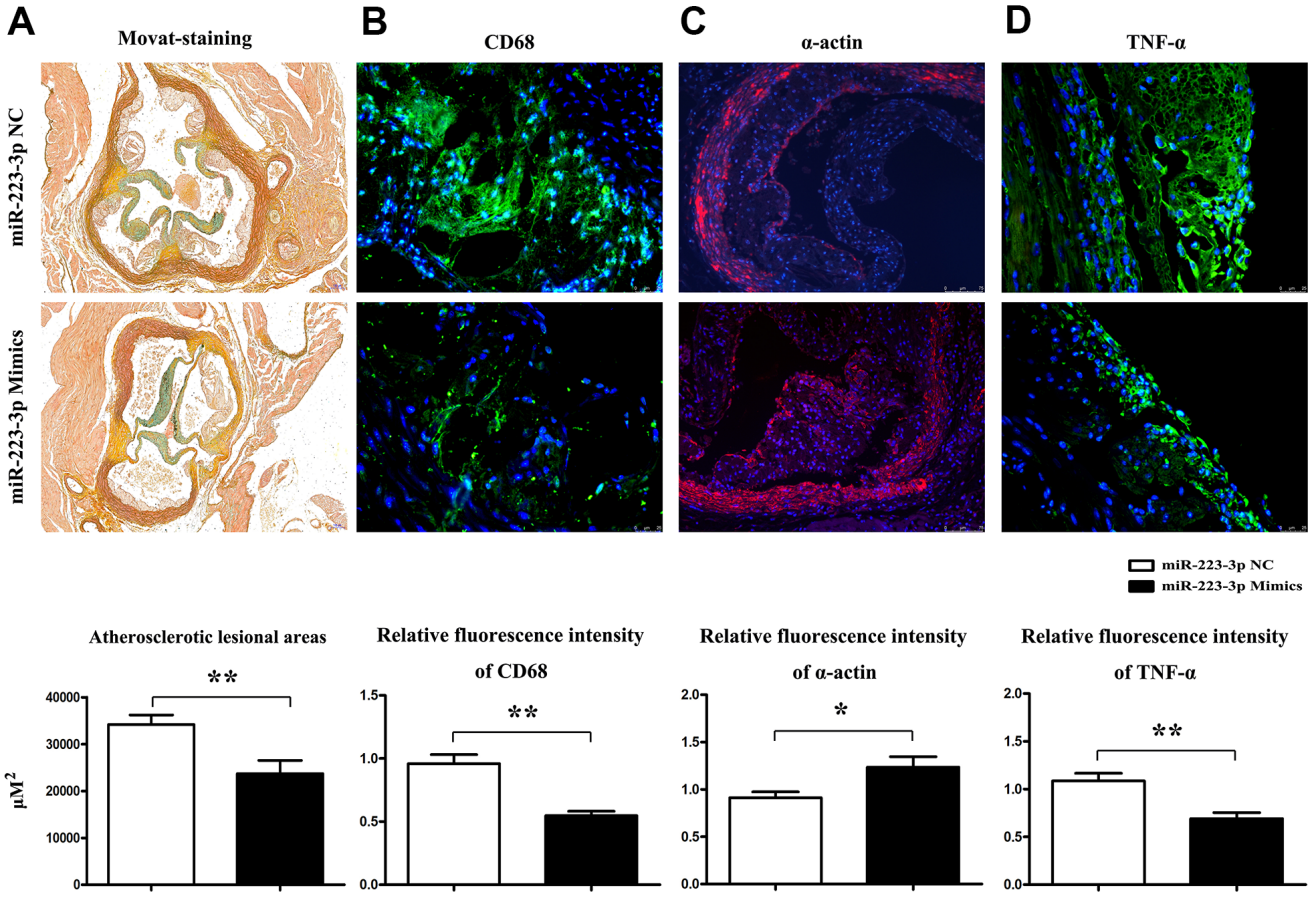
**Prevention of AS progression by miR-223-3p mimics.** (**A**) Representative Movat staining of the histopathology of mice's carotid roots and a pronounced decrease in the extent of atherosclerotic lesions in the carotid roots tissues from miR-223-3p mimics. (*P<0.05). (**B**) The plaque area staining positive for macrophages (CD68) in the miR-223-3p mimics group and NC group (*P<0.05). (**C**) The SMA staining in mice's aortic roots in the miR-223-3p mimics group and NT group (*P<0.05). (**D**) TNF-α staining in mice's aortic roots in the miR-223-3p mimics group and NT group(*P<0.05).

To functionally investigate the role of miR-223-3p in vulnerable atherosclerotic lesions, we applied the well-established mice atherosclerosis HFDs, in which atherosclerotic lesions were induced in apolipoprotein-E deficient (ApoE^-/-^) mice by a high-fat diet. To further elucidate the *in vivo* effects of miR-223-3p in AS, ApoE^-/-^ mice were assigned to high-fat diet (HFD) + miR-223-3p negative control (NC), and HFD + miR-223-3p mimics. Mice carotid roots and myometrial tissues were obtained and stained with Movat staining. Representative staining results were shown in Figure 2A, atherosclerotic lesions were shown in both groups, and an obvious decrease in the extent of atherosclerotic lesions in the miR-223-3p mimics. Moreover, the plaque area staining positive for macrophages (CD68+) in miR-223-3p mimics decreased compared with the NC group, whereas the SMA staining level was increased in miR-223-3p mimics (Figure 2B, 2C), suggesting that miR-223-3p mimics could reduce macrophages accumulation and promote smooth muscle cells composition. ELISA results revealed that the TNF-a immunopositive plaque area also reduced in miR-223-3p mimics compared with the NC group (Figure 2D). The quantitative analysis was consistent with the above findings obtained in our *in vitro* experiments. (P<0.05, miR-223-3p mimics vs. miR-223-3p NC). In brief, these *in vivo* experimental data implied that miR-223-3p mimics could prevent AS from developing.

### Targeting relationship between miR-223-3p and MEK/ERK

**Figure 3:**
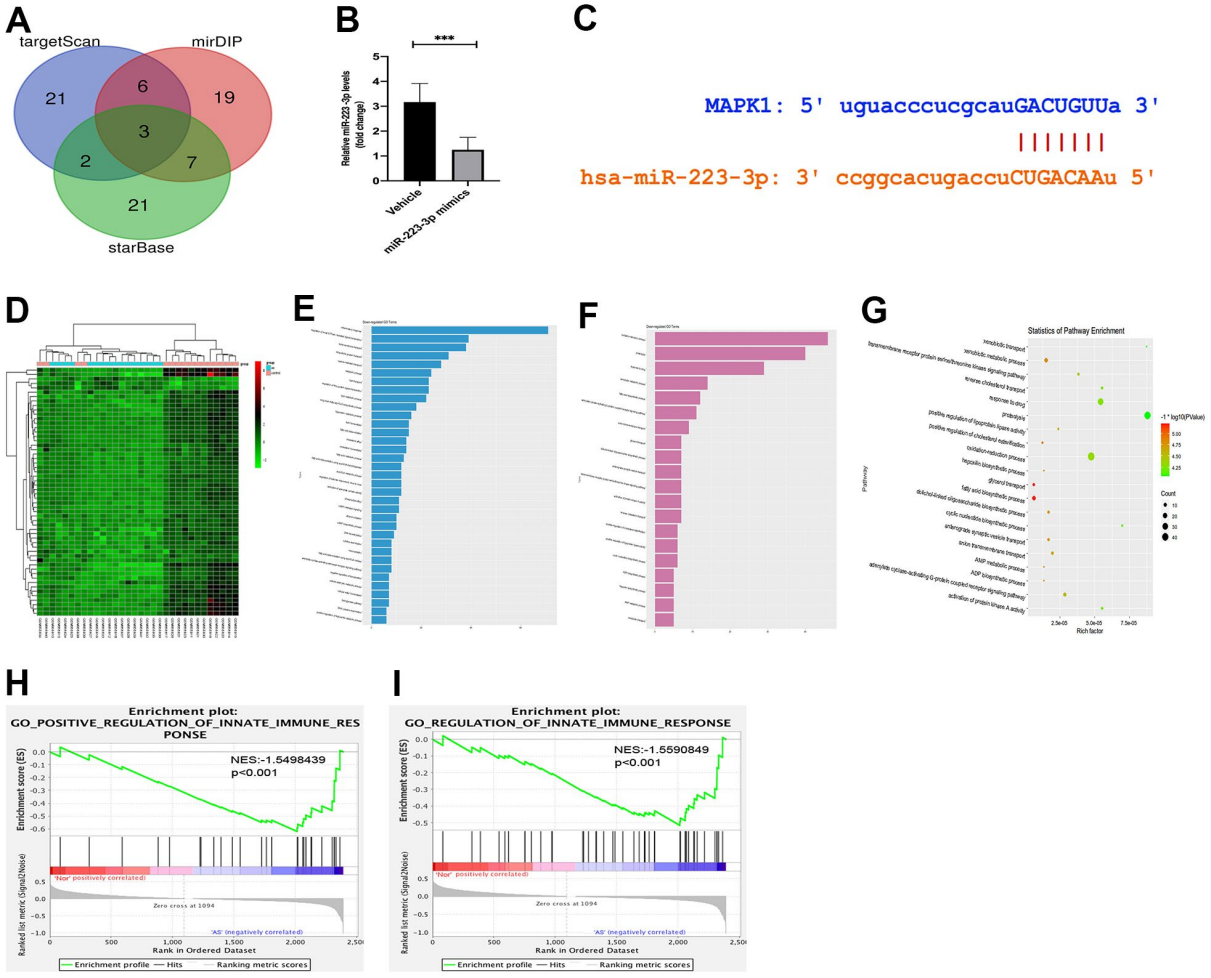
**Targeting relationship between miR-223-3p and MEK/ERK.** (**A**) The miRNA target prediction databases exposed three potential genes (PLAGL2, FLOT2, and MAP2K1) of miR-223-3p. (**B**) The qPCR revealed that MAP2K1(MEK1) expression was noticeably decreased in macrophages transfected with miR-223-3p mimics. (**C**) miRDIP and starBase databases showed that miR-223-3p and MAP2K1 had targeted binding regions. (**D**) The hierarchical clustering analysis exhibited the distinction on differentially expressed genes in the heat map. (**E**) Go enriched up-regulated pathways. (**F**) Go enriched down-regulated pathways. (**G**) The relevant partial results for KEGG pathways. (**F**) The gene set enrichment analysis (GSEA) revealed that the innate immune pathway regulation was functionally enriched in AS. (**H**, **I**) GSEA revealed that the innate immune pathway regulation was functionally enriched in AS.

Given that miRNAs exert their function by reducing their downstream expression of target genes [24]. The variety of functions of miR-223 has been linked to the suppression of many different target genes in inflammatory pathologies [25, 26]. Therefore, miR-2233p may affect cell differentiation and activation by suppressing its target genes, which have been protective against inflammatory response. We obtained three potential genes (PLAGL2, FLOT2, and MAP2K1) of miR-223-3p by combining the results from the online miRNA target prediction databases (Figure 3A). To confirm whether the three potential genes were the downstream target genes for miR-223-3p, we examine the expression of the three potential genes in macrophages transfected with miR-223-3p mimics. The qPCR results revealed that MAP2K1(MEK1) expression was noticeably decreased in macrophages transfected with miR-223-3p mimics (Figure 3B). Furthermore, miRDIP and starBase databases were applied, and we found miR-223-3p and MAP2K1 had targeted binding regions (Figure 3C). There results suggested that MAP2K1 was a direct target of miR-223-3p. Upon activation, Raf mediates phosphorylation of MEK1 and MEK2, which in turn phosphorylate ERK1 and ERK2 [27]. Thus, we hypothesize that the regulation of miR-223-3p may be through the MEK/ERK signaling pathway in atherosclerosis. To assess the inflammatory response signaling pathway in AS, we downloaded the GSE34822 dataset from the GEO database, comprised of atherosclerosis samples. As shown in Figure 3D, 158 up-regulated differentially expressed genes (DEGs) and 206 down-regulated DGEs were obtained using the criteria of Padj < 0.01, |log2FC| > 1.2, hierarchical clustering analysis exhibited the distinction on differentially expressed genes in the heat map. We then performed pathway analysis by David and established Go enriched up-regulated pathways (Figure 3E), including inflammatory response, metabolic process, lipid transport, and intracellular protein transport. We also found down-regulated pathways, including anion transmembrane transport, glycerol transport, and ADP biosynthetic process. (Figure 3F) Correspondingly, relevant partial results for KEGG pathways were exhibited in Figure 3G. Furthermore, the gene set enrichment analysis (GSEA) revealed that the innate immune pathway regulation was functionally enriched in AS (Figure 3H, 3I). Thus, we pick up miR-223-3p, MEK/ERK1/2, and the inflammatory response pathway for further investigation.

### Regulation of inflammatory cytokine secretion and p-ERK1/2 by miR-223-3p

**Figure 4:**
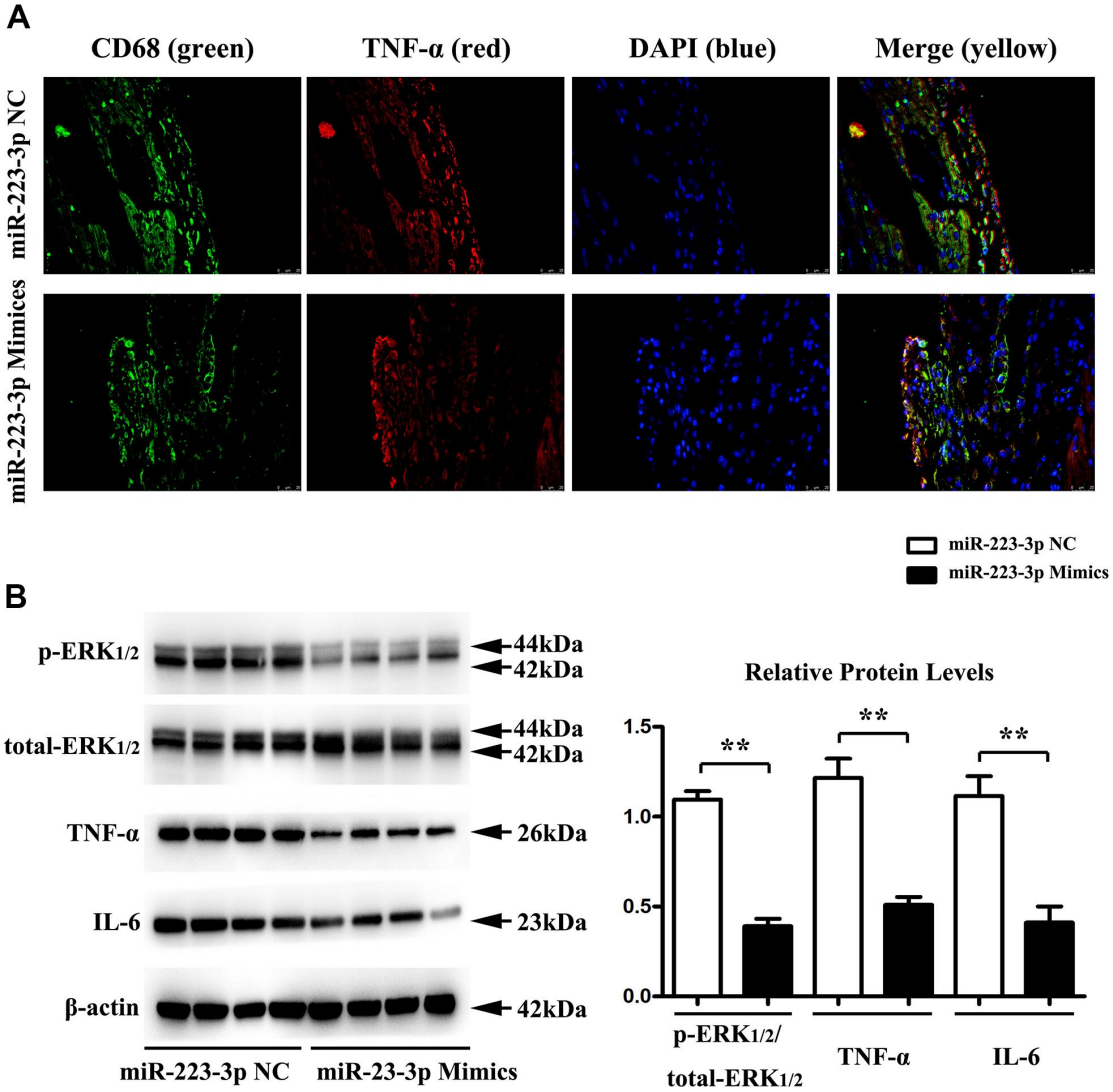
**Regulation of inflammatory cytokine secretion and p-ERK1/2 by miR-223-3p.** (**A**) Double-immunofluorescence revealed significant co-localization of the macrophage marker CD68+ and TNF-α in carotid tissues from 223-3p mimics and. (**B**) The western blots analysis revealed significantly decreased expression levels of IL-6, TNF-a, and p-ERK1/2 in carotid tissues from the miR-223-3p mimics group. The quantitative analysis of relative protein expression exposed the associated decrease in the protein levels of IL-6, TNF-a, and ERK1/2 in carotid tissues of miR-223-3p mimics (*P<0.05).

Double-immunofluorescence was performed on carotid tissues from 223-3p mimics and the NC mice group. As shown in Figure 4A, the double-immunofluorescence exhibited significant co-localization of the macrophage marker CD68+ and TNF-α, suggesting that the macrophages express TNF-α in miR-223-3p mimics (Figure 4A). To further investigate the possible role of miR-223-3p in the inflammatory response of AS, protein expression changes in mice carotid tissues were determined by quantitative analysis and western blots, respectively. We found significantly decreased expression levels of inflammatory cytokine (IL-6 and TNF-a) in carotid tissues from the miR-223-3p mimics group in western blots (Figure 4B). Additionally, we also found significantly reduced p-ERK1/2 expression level in miR-223-3p mimics. (Figure 4B). The quantitative analysis of relative protein expression confirmed the associated decrease in the protein levels of IL-6, TNF-a, and ERK1/2 in carotid tissues of miR-223-3p mimics as compared with the NC group (Figure 4B, P<0.05), suggesting that miR-223-3p mimics could repress inflammatory response and phosphorylation ERK1/2 in AS.

### MiR-223-3p mimics inhibits the inflammatory response via the ERK1/2 pathway *in vitro*

**Figure 5:**
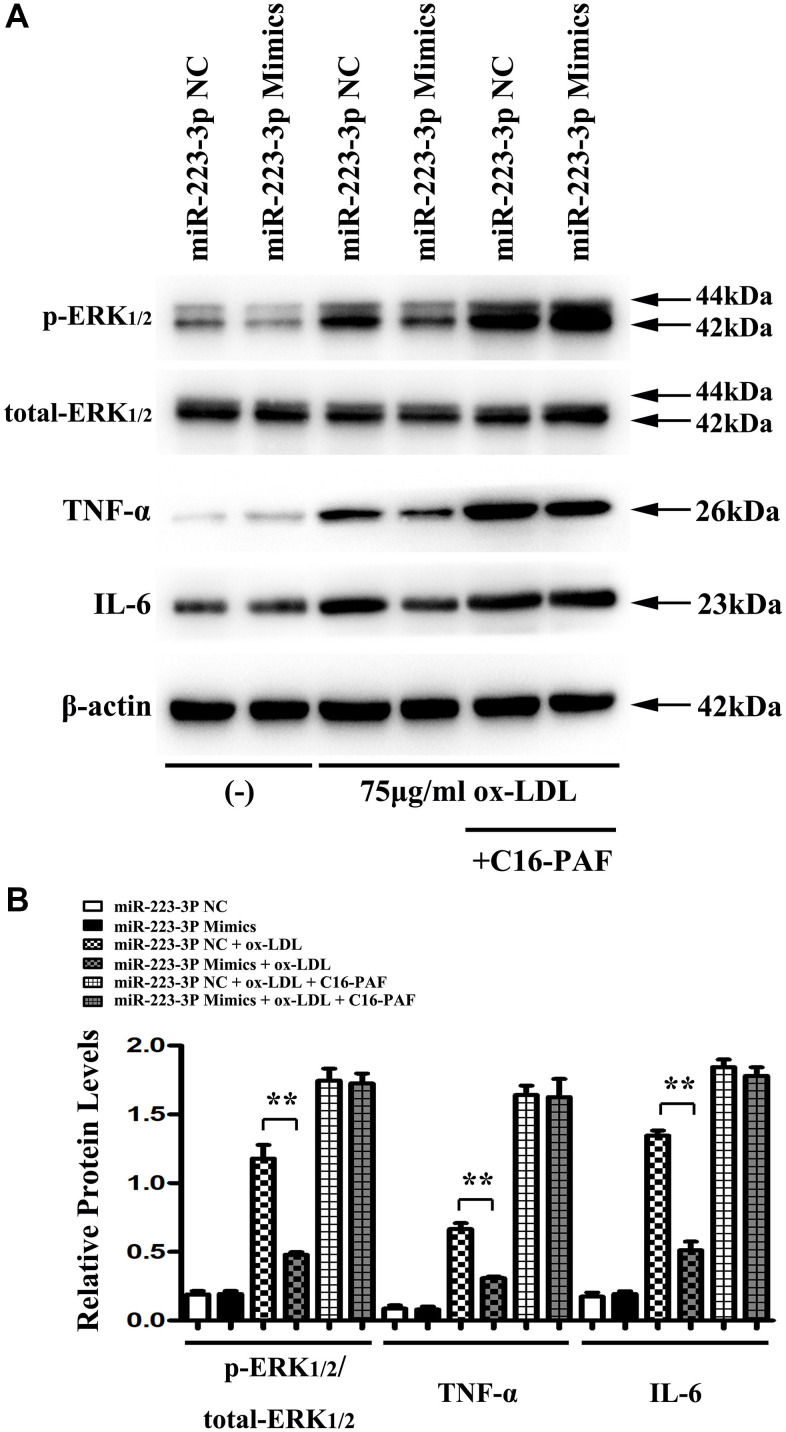
**MiR-223-3p mimics inhibits the inflammatory response was via the ERK1/2 pathway *in vitro*.** (**A**) The western blots analysis revealed that p-ERK1/2, IL6, and TNF-a were significantly suppressed in miR-223-3p mimic-transfected macrophages with ox-LDL stimulation, and the decreased expression levels of p-ERK1/2, IL6, and TNF-a were markedly enhanced in miR-223-3p mimic-transfected macrophages with ox-LDL by C16-PAF. (**B**) The quantitative analysis revealed that the relative protein levels of p-ERK1/2, IL6, and TNF-a were decreased in miR-223-3p mimic-transfected macrophages with ox-LDL stimulation, and the decreased relative protein levels of p-ERK1/2, IL6, and TNF-a were markedly enhanced in miR-223-3p mimic-transfected macrophages with ox-LDL by C16-PAF. (*P<0.05).

The macrophages might initiate and exacerbate inflammation with ox-LDL stimulation and prompt the destabilization of the atherosclerotic plaques [28]. To investigate the biological importance of ERK1/2 as a target of miR-223-3p, miR-223-3p mimic/NC-transfected macrophages were stimulated with ox-LDL and saline to detect the ERK1/2, IL6, TNF-a expression in *in vitro*. Using western blots, we found that the expression levels of p-ERK1/2, IL6, and TNF-a were significantly suppressed in miR-223-3p mimic-transfected macrophages with ox-LDL stimulation as compared with miR-223-3p NC-transfected macrophages with ox-LDL (Figure 5A). The result of *in vitro* implied that miR-223-3p mimic could repress the inflammatory response in AS. To further determine whether miR-223-3p mimic mediated inflammatory effect in ox-LDL stimulated macrophages was via ERK1/2 signaling pathway, C16-PAF, an ERK1/2 agonist was injected into the culture medium of the miR-223-3p mimic/NC-transfected macrophages with ox-LDL stimulation. Interestingly, we found that the decreased expression levels of p-ERK1/2, IL6, and TNF-a in miR-223-3p mimic-transfected macrophages with ox-LDL were markedly reversed by C16-PAF, suggesting that miR-223-3p mimic mediated inflammatory effect in ox-LDL stimulated macrophages was via ERK1/2 signaling pathway (Figure 5A). As shown in Figure 5B, the quantitative analysis of relative protein expression was consistent with our western blot analysis data (P<0.05, Figure 5B). Therefore, these data suggested the essential role for ERK1/2 as a mediator of the biological effects of miR-223-3p-mediated inflammatory effect in AS.

## DISCUSSION

AS is an inflammatory diseases, the macrophages play a significant roles in the formation and progression of atherosclerosis, therefore the factors regulated the inflammatory factors and associated modulatory pathway proteins secreted by lesional macrophages, deserved of research [29, 30]. Previous studies demonstrated the miRNAs involved in atherosclerotic progression and also played crucial effects in modulating lesional inflammatory responses [31–33]. In the current study, Firstly, bioinformatics analysis were used for screening out the miRNA-223-3p involved in the progression of AS, the expression level of miR-223-3p was up-regulated in both carotid arteries and serum of AS patients, the miR-223-3p is associated with a higher overall survival rate, suggesting that the miR-223-3p could be an excellent diagnostic marker for AS, data shown in Figure 1A–1C. And further the Q-PCR were used for testing the mRNA levels of miR-223-3p in human species of stable and vulnerable atherosclerotic plaques, shown in Figure 1E. Besides, Fluorescent *In Situ* Hybridization (FISH) assay revealed miR-223-3p co-localization with CD68+ macrophages in vulnerable atherosclerotic lesions of AS patients, as showed in Figure 1F, thereby the macrophages were elected for target cellular type in our study.

The disease based on atherosclerosis, such as myocardial infarction, apoplexia, cerebral infarction, obliterans of the Lower Extremities and et al., attracted from youth to middle aged and old aged people [34, 35]. The vulnerable/ instable plaques mainly appeared in old persons [36] and in this study, the atherosclerotic lesions from patients of the first affiliated hospital of Hebei medical university were obtained by carotid endarterectomy (CEA). Consequently, we carried out a histological analysis of aortic sections from AS patients to evaluate the morphological differences, representative Movat staining results for vulnerable plaque and stable plaque were exhibited in Figure 1D, and we found decreased miR-223-3p expression level in the vulnerable plaque compared with stable plaque, indicating that miR-223-3p has the value to be made further research on. The detailed mechanisms of miR-223-3p were further verified in animal models, we applied the well-established atherosclerotic mouse models, apolipoprotein-E deficient (ApoE-/-) mice, in which AS lesions were induced by fed with high fat diet. ApoE^-/-^ mice were treated with miR-223-3p negative control and mimic and pathological analysis results showed that the increase of miR-223-3p decreased lesional areas following with macrophage decrease, TNF-α secretion and smooth muscle cells increase. Lesional macrophages is the major cellular resource responding for inflammatory factors secretion, TNF-α, IL-6, IFN-γ, et al., resulting in accumulating circulating monocytes and macrophages into AS and promoting outbreak of inflammatory response [37–39], thus forming a vicious cycle; and the lesional SMC is the major cell secreting collagen, extracellular matrix components, the principal contents of fibrotic caps, protected the vulnerable plaques from rupture, playing protective roles [40, 41]. Our vivo experimental data illustrated that miR-223-3p mimics-mediated inhibition of inflammatory responses is one of the mechanisms involved in the development of AS.

MiRNAs exert their function by reducing their downstream expression of target genes. The variety of functions of miR-223 has been related to the suppression of many different target genes in inflammatory pathologies [25]. Therefore, miR-223-3p may affect cell differentiation and activation by suppressing its target genes, which have been protective against inflammatory response. We obtained the potential gene MAP2K1 of miR-223-3p by combining the results from the online miRNA target prediction databases. The qPCR results revealed that MAP2K1(MEK1) expression was noticeably decreased in macrophages transfected with miR-223-3p mimics. Furthermore, miRDIP and starBase databases were applied, miR-223-3p and MAP2K1 had targeted binding regions. There results suggested that MAP2K1 was a direct target of miR-223-3p. Upon activation, Raf mediates phosphorylation of MEK1 and MEK2, which in turn phosphorylate ERK1 and ERK2 [27]. Thus, we hypothesize that the regulation of miR-223-3p may be through the MEK/ERK signaling pathway in atherosclerosis. To assess the inflammatory response signaling pathway in AS, we downloaded the GSE34822 dataset from the GEO database, comprised of atherosclerosis samples. The hierarchical clustering analysis exhibited the distinction on differentially expressed genes in the heat map according to the GSE34822 dataset. We then performed pathway analysis by David and established Go enriched up-regulated and down-regulated pathways and relevant partial results for KEGG pathways. The gene set enrichment analysis (GSEA) revealed that the innate immune pathway regulation was functionally enriched in AS. Thus, we pick up miR-223-3p, MEK/ERK1/2, and the inflammatory response pathway for further investigation.

Double-immunofluorescence exhibited significant co-localization of the macrophage marker CD68+ and inflammatory factor TNF-α, indicating the TNF-α secreted by macrophage in AS lesions. To further investigate the possible role of miR-223-3p in the inflammatory response of AS, protein expression changes in mice carotid tissues were determined by quantitative analysis and western blots. Respectively, we found significantly decreased expression levels of inflammatory cytokine (IL-6 and TNF-a) in carotid tissues from the miR-223-3p mimics group compared with the NC group. Evidence has indicated that the MAPK1 signaling pathway is involved in miR-223-3p inhibition by combining with 3’-UTR of MAPK1 for further degradation [23, 42]. As the MAPK signals especially the MAPK1/ERK2 is the major modulatory signal proteins for inflammatory factors synthesis and sections [43, 44]. Using quantitative analysis and western blots, we found a significantly decreased p-ERK1/2 expression level in miR-223-3p mimics compared with the NC group (P<0.05), suggesting that miR-223-3p mimics could repress inflammatory response and phosphorylation ERK1/2 in AS, these results were consistent with our bioinformatics analysis.

The macrophages might initiate and exacerbate inflammation with ox-LDL stimulation and prompt the destabilization of the atherosclerotic plaques [28]. Using western blots analysis, we found that the expression levels of p-ERK1/2, IL6, and TNF-α were significantly suppressed in miR-223-3p mimic-transfected macrophages with ox-LDL stimulation as compared with miR-223-3p NC. C16-PAF, an ERK1/2 agonist, was injected into the culture medium of the miR-223-3p mimic/NC-transfected macrophages with ox-LDL stimulation to investigate whether miR-223-3p mimic mediated inflammatory effect was via ERK1/2 signaling pathway. Interestingly, we found that the decreased expression levels of p-ERK1/2, IL6, and TNF-a were markedly enhanced in miR-223-3p mimic-transfected macrophages with ox-LDL by C16-PAF, suggesting that miR-223-3p mimic mediated inflammatory effect was via ERK1/2 signaling pathway. These results implied that the MEK/ERK1/2 signaling pathway was necessary to facilitate the development of miR-223-3p in AS. Our *in vitro* experiment demonstrated that miR-223-3p could play an anti-atherosclerosis role by modulating ERK activity and inflammatory factor secretion.

In conclusion, our study elucidates that miR-223-3p prevents the development of AS and reduced inflammatory response through down-regulation of MEK1/ERK1/2, which suggests that miR-223-3p /MEK1/ERK1/2 can be potentially used as an attractive therapeutic for AS.

## MATERIALS AND METHODS

### Patients and specimens

8 patients with stable plaques n=4, and vulnerable plaques n=4 underwent diagnostic carotid CT and ultrasonic testing to intent to revascularize the culprit arteries shown in Figure 1B. All patients presented with atheromatous plaques of the extracranial artery but without thrombus, the information of risk factors for atherosclerosis such as diabetes and hypertension were also collected. The detailed patient information were collected.

### Establishment of mice AS models

The ApoE^−/−^ mice (C57Bl/6 background) were obtained from Beijing HFK Biotechnology Co., Ltd (Beijing, China). The animal use protocol for this study has been reviewed and approved by the Laboratory Animal Ethical Committee, Hebei Medical University first Affiliated Hospital. ApoE^−/−^ mice established adeno-associated virus serotype-9 (AAV9) mediated miR-223-3p overexpression via the tail vein. The groups were set as follows: high-fat diet (HFD) + miR-223-3p negative control (NC), and HFD + miR-223-3p mimics groups via tail vein injection. The mice were fed a high-fat diet (HFD) to induce early atherosclerotic lesions. After providing a high-fat diet for two months and transfection with AAV9, the mice were sacrificed.

### Enzyme-linked immunosorbent assay

After injection with pentobarbital sodium, blood samples were gathered from the carotid artery of AS patients and controls. The serum contents were treated with regional citrate anticoagulation (RCA) and centrifuged at 2500g for 20 min at four ° C. Blood cells were recovered by centrifugation at 700 × g for 20 min at 4° C. The ELISA kit (Cat. No. 210-A-050, R&D Systems, Minneapolis, MN) was conducted to identify the plasma and macrophages expressed according to the kit instructions. The serum levels of inflammatory cytokines interleukin-6 (IL-6) (eBioscience) and tumor necrosis factor-α (TNF-α) (eBioscience) were examined using ELISA kits (RapidBio, CA, USA).

### Movat-staining

After thoroughgoing washing with water, the aortic tissues were rinsed by 60% isopropanol for 5 min and stained with the Movat-staining dyeing solution for 2 h, then differentiated and rinsed by 60% isopropanol for 6~7 times. Finally, the images were captured under an optical microscope (×400, Olympus Optical Co., Ltd.).

### Macrophage culture and transfection

Mouse macrophage-like cell line RAW 264.7 was obtained from Peking Union Medical College. After adding Dulbecco's modified eagle's medium (DMEM, General Electric Company, Utah, USA) with 10% fetal bovine serum (FBS) (Gibco Company, Grand Island, NY, USA), the cells were cultured at 37° C with 5% CO2. Forty-eight hours later, the cells were treated with 0.25% trypsin (Biotime Biotechnology) and re-cultured in a 75 mL flask. The cells were seeded in 6-well plates at 2.5 × 105 cells per well and cultured in a medium before transfection. According to the manufacturer's instructions, cells were then transfected using Lipofectamine 2000 (Santa Cruz Inc., Santa Cruz, CA, USA). The miRNA and Lipofectamine 2000 reagent were individually diluted to 20% in serum-free DMEM and incubated for 10 mins. Then cultured macrophages were transfected with miR-223-3P mimics, miR-223-3P mimics negative control (NC). The primary culture medium was detached three hours after transfection, and the cells were cultured for an additional 24 hours in the transfection complex. Twenty-four hours after transfection, the culture medium was removed, and the cells were re-cultured for an extra 48 hours. Consecutively diluted ox-LDL at 50, 100, and 200 mg/L were used to treat the cells for 12 h, 24 h, and 48 h. qPCR was conducted on a Step One Plus system and Chromo4 using specific primers (Applied Biosystems, Foster City, CA). Values of miR-223-3P were normalized (cultured macrophages) and presented as 2−ΔΔCT equation through boxplot with smallest and largest whiskers.

### Fluorescent *In Situ* Hybridization (FISH) assay

The FISH assay was applied to detect the location and expression of miR-223-3P in aortic tissues. The aortic tissues were fixed with 4% paraformaldehyde at room temperature and embedded in paraffin (4-μm thickness). Subsequently, the tissues were deparaffinized by mineral oil. They rehydrated in graded dilutions of ethanol (100%, 100%, 90%, 80%, 70%, 50%), digested with 16 μg of proteinase K (Thermo Fisher Scientific, San Jose, CA, USA) at 37° C for 15 min, rinsed in 0.2% glycine in PBS (Sigma-Aldrich) for 10 min, fixed with 4% paraformaldehyde phosphate-buffered saline (PBS) at room temperature for 10 min. Then, paraffin slides were hybridized with fluorescent (Cy3-labeled) oligonucleotide miR-223-3P probes (5′-UGUCAGUUUGUCAA AUACCCC-3′) at 37° C overnight. After probe hybridized, tissues were incubated in extraction buffer (20 mM Tris-HCl, pH7.5, 2 mM EDTA, 0.25% SDS, 0.225M NaCl), at 37° C for 20 min. Tissues were stained with CD68+ with purified ab81289 (Abcam Inc., Cambridge, MA) at 1:200. The conventional heat-mediated antigen retrieval was conducted with Tris-EDTA buffer (pH 7.6, Abcam Inc., Cambridge, MA). Goat Anti-Rabbit IgG H&L (HRP) was used as the secondary antibody at 1/5000 dilution. 4,6-diamidino-2-phenylindole (DAPI) staining was used for cell nuclear counterstain with laser scanning confocal microscopy (Olympus, Japan). A light microscope carried out the qualitative analysis ((Nikon Corporation, Tokyo, Japan). Images of the sections were captured by fluorescence microscope (magnification, ×200; BX50, Olympus) and analyzed using Image-Pro Plus 6.0 software (Media Cybernetics, USA).

### Western blot analysis

The protein extracts from aortic tissues and cells were denatured. The protein samples were separated by SDS-PAGE (10% gel) and transferred to PVDF membranes (Millipore, MA, USA). After washing with Tris-buffered saline with Tween- 20 (TBST), the protein samples were incubated with primary and secondary antibodies (Abcam) for 1h. Band intensities were detected by an Odyssey infrared imaging system (Li-cor, Lincoln, NE, USA).

### RNA isolation and real-time PCR

Total RNA was isolated from macrophages and aortas using a Trizol reagent (Life Technologies, Inc, Burlington, ON, Canada) and purified by the RNA Easy kit (Qiagen Inc., Valencia, CA). qPCR fluorophore SYBR-Green was purchased from Beijing Solarbio Science and Technology Co., Ltd. Reverse transcription-quantitative PCR (qRT-PCR) of the target mRNA was carried out using primers, The mice primers: miR-223-3p’ F 5'-GTG CAG GG TCC GAG GT-3';R 5'-CGG GCT GTC AGT TTG TCA-3'; U6’ F 5'-CTC GCT TCG GCA GCA CA-3'; R 5'-AAC GCT TCA CGA ATT TGC GT-3'. human primers : miR-223-3p F 5’-ACA CTC CAG CTG GGT GTC AGT TTG TCA AAT-3’; R 5’-CTC AAC TGG TGT CGT GGA GTC GGC AAT TCA GTT GAG TGG GGT AT-3’; U6 F 5’- CGC TTC GGC AGC ACA TAT AC -3’; R 5’- AAA TAT GGA ACG CTT CAC GA -3’. Changes in gene expression levels of >2.5-fold were considered significant.

### Statistical analysis

All data were expressed as mean ± standard deviation (SD). Statistically significant differences among groups were evaluated by one-way analysis of variance (ANOVA) or Student's t-test using SPSS 20.0 software. Statistical significance was signified when p values <0.05.

### Data availability

The data used to support the findings of this study are included within the article.

### Ethics approval

The animal use protocol for this study has been reviewed and approved by the Laboratory Animal Ethical Committee, Hebei Medical University Second Affiliated Hospital.

### Availability of data and materials

The datasets used and/or analyzed during the current study are available from the corresponding author on reasonable request.
